# Investigating the role of a *Tannerella forsythia* HtrA protease in host protein degradation and inflammatory response

**DOI:** 10.3389/froh.2024.1425937

**Published:** 2024-07-05

**Authors:** Susanne Bloch, Fiona F. Hager-Mair, Johanna Bacher, Markus B. Tomek, Bettina Janesch, Oleh Andrukhov, Christina Schäffer

**Affiliations:** ^1^ Competence Center for Periodontal Research, University Clinic of Dentistry, Medical University of Vienna, Vienna, Austria; ^2^ NanoGlycobiology Research Group, Department of Chemistry, Institute of Biochemistry, Universität für Bodenkultur Wien, Vienna, Austria; ^3^ Department of Biotechnology, Institute of Bioprocess Science and Engineering, Universität für Bodenkultur Wien, Vienna, Austria

**Keywords:** red complex bacterium, recombinant protease, E-cadherin degradation, inflammatory mediators, human gingival fibroblasts, macrophages, virulence factor, periodontitis

## Abstract

**Introduction:**

Degradation of host proteins by bacterial proteases leads to the subversion of the host response and disruption of oral epithelial integrity, which is considered an essential factor in the progression of periodontitis. High-temperature requirement A (HtrA) protease, which is critical for bacterial survival and environmental adaptation, is found in several oral bacteria, including the periodontal pathogen *Tannerella forsythia*. This study investigated the proteolytic activity of HtrA from *T. forsythia* and its ability to modulate the host response.

**Methods:**

HtrA of *T. forsythia* was identified bioinformatically and produced as a recombinant protein. *T. forsythia* mutants with depleted and restored HtrA production were constructed. The effect of *T. forsythia* wild-type, mutants and recombinant HtrA on the degradation of casein and E-cadherin was tested *in vitro*. Additionally, the responses of human gingival fibroblasts and U937 macrophages to the different HtrA-stimuli were investigated and compared to those triggered by the HtrA-deficient mutant.

**Results:**

*T. forsythia* wild-type producing HtrA as well as the recombinant enzyme exhibited proteolytic activity towards casein and E-cadherin. No cytotoxic effect of either the wild-type, *T. forsythia* mutants or rHtrA on the viability of host cells was found. In hGFB and U937 macrophages, both *T. forsythia* species induced an inflammatory response of similar magnitude, as indicated by gene and protein expression of interleukin (IL)-1β, IL-6, IL-8, tumour necrosis factor *α* and monocyte chemoattractant protein (MCP)-1. Recombinant HtrA had no significant effect on the inflammatory response in hGFBs, whereas in U937 macrophages, it induced a transient inflammatory response at the early stage of infection.

**Conclusion:**

HtrA of *T. forsythia* exhibit proteolytic activity towards the host adhesion molecule E-cadherin and has the potential to influence the host response. Its role in the progression of periodontitis needs further clarification.

## 1 Introduction

Periodontitis is one of the most prevalent microbially-induced disorders worldwide (1). The primary cause of this inflammatory disease, which affects the tissues supporting the teeth, is the presence of a dysbiotic microbial community in the subgingival crevice (2). In a healthy state, subgingival biofilms are formed predominantly by commensal microbes; however, periodontal pathogens may elevate the pathogenicity of the whole community and trigger an exacerbated inflammatory response that leads to tissue destruction and ultimately tooth loss (3, 4). Periodontitis is linked to numerous systemic conditions, such as rheumatoid arthritis, cardiovascular disease, diabetes, cancer and Alzheimer's disease (5–7). The microbial aetiology of periodontal disease has long been established, and among the periodontal pathogens, three species are most often associated with disease development—*Porphyromonas gingivalis*, *Tannerella forsythia* and *Treponema denticola* (8, 9). These bacteria employ an array of virulence factors to establish their pathogenicity within the oral cavity (10, 11), including several proteolytic enzymes. In the case of *P. gingivalis*, gingipains—aggressive cysteine proteases (RpA, RgpB, and Kgp)—are attaining increasing interest for their wide-reaching effects on the host immune system and systemic diseases (12). For example, they exert proteolysis at the cell surface of human gingival epithelial cells (13) and enhance blood-brain barrier permeability (14). Similarly, for *T. forsythia*, a plethora of proteases has been linked to the bacterium's pathogenicity, including, *e.g.*, PrtH proteases transferring attachment loss (15), a trypsin-like cysteine protease with both arginine- and citrulline-specific activities (16), and secretory KLIKK proteases exhibiting activity towards diverse protein substrates, including collagen, gelatine, elastin and casein (17).

A less-investigated class of proteases in oral pathogens are HtrA (high-temperature requirement A) enzymes. For pathogens, HtrA family members are generally crucial for survival, adaptation to environmental changes, and tolerance of harsh conditions such as elevated temperatures (heat-shock response), extreme pH and oxidative or osmotic stress (18). In several cases, loss of HtrA function correlates with reduced virulence and restricted bacterial growth under external stresses (19). Thus, bacterial HtrAs are regarded as promising candidates for developing novel antibacterial strategies (20). Mechanistically, HtrAs have ATP-independent dual chaperone-protease activity and mediate protein quality control (19, 21). Members of the HtrA protein family consist of a conserved chymotrypsin-like serine protease domain with the catalytic triad composed of a histidine, aspartate and serine residue (22), and at least one C-terminal regulatory PDZ (post synaptic density of 95 kDa, discs large, and Zonula Occludens 1) domain for substrate recognition, binding and oligomerization (21, 22). The two major structural subdivisions within this protein family are the DegP/O and DegS enzymes, which ensure protein quality control in the periplasm of *E. coli* (21). In addition to the serine-protease domain, they contain either two PDZ domains and an N-terminal signal peptide for cytoplasmic membrane translocation (DegP/O), or a single PDZ domain preceded by an N-terminal transmembrane segment for membrane anchoring (DegS) (18). Other HtrA proteins can be translocated to the cell surface or secreted, enabling them to participate in bacterial colonization or host invasion (22, 23). It is proposed that the extracellular activity of HtrAs involves their transport through outer membrane vesicles (22).

HtrA enzymes are crucial for the virulence of several important pathogens due to their diverse functionalities. These pathogens include *E. coli* (24), *Listeria monocytogenes* (25), *Legionella pneumophila* (26), *Borrelia burgdorferi* (27), *Helicobacter pylori* (28), *Campylobacter jejuni* (29), and *Bacillus anthracis*, among others. In *H. pylori*, HtrA acts as a secreted virulence factor that cleaves the ectodomain of the cell-adhesion protein E-cadherin leading to E-cadherin shedding and disruption of the epithelial barrier functions, allowing *H. pylori* to access the intercellular space (30). In *C. jejuni*, secreted HtrA facilitates transepithelial migration by degrading E-cadherin (31). A *C. jejuni* deletion mutant lacking HtrA was shown to induce lower levels of apoptosis and reduced secretion of the pro-inflammatory cytokines monocyte chemoattractant protein (MCP)-1, interleukin (IL)-6, tumour necrosis factor (TNF)-α, and interferon (IFN)-γ in a mouse infection model, underscoring the protease's role in the bacterium's virulence (32). Recently, it was found that surface-bound HtrA of *C. jejuni*, but not secreted HtrA, disrupts epithelial cell-to-cell junctions (33). In *B. anthracis*, HtrA plays a regulatory role, influencing expression of more than 1,000 genes under stress (34). A *B. anthracis* Δ*htrA* deletion mutant was unable to proliferate in macrophages and impaired their lysis (35). In the context of periodontitis, in *P. gingivalis*, HtrA was shown to regulate gingipain activity and adaptation to oxidative and long-term heat stress (36, 37). The protease also influenced bacterial survival in a mouse model, and a *P. gingivalis* Δ*hrtA*-deletion mutant displayed cell-dependent effects on invasiveness compared to the *P. gingivalis* W83 parent wild-type strain, with increased invasion of epithelial cells, while invasion of endothelial cells was unaffected (37). These findings suggest a prominent role of HtrA in the interaction of this periodontal pathogen with the host.

In this study, we obtained evidence that *T. forsythia* ATCC 43037 produces a proteolytically active HtrA ortholog. We identified the *htrA* gene in *T. forsythia* ATCC 43037, produced the predicted HtrA recombinantly in *E. coli* cells, and demonstrated proteolytic activity of the *T. forsythia* enzyme on different substrates *in vitro*. Furthermore, we constructed a *T. forsythia* Δ*htrA* deletion mutant. Using this mutant along with the *T. forsythia* parent strain and recombinant HtrA, we conducted infection studies to assess the enzyme’s effect on cellular viability and pro-inflammatory cytokine production in human gingival fibroblasts and macrophages.

## 2 Materials and methods

### 2.1 Bacterial strains and cultivation conditions

*T. forsythia* ATCC 43037 (American Type Culture Collection-ATCC), the corresponding *htrA* deletion mutant (*T. forsythia* Δ*htrA*) and the back-complemented mutant (*T. forsythia* Δ*htrA*^+^)-for construction of mutants see 2.1.1 and 2.1.2-were grown anaerobically at 37 °C for 5 days in brain–heart infusion broth (BHI) (Oxoid) supplemented with MurNAc (20 µg/ml), horse serum (5%), and 50 µg/ml gentamycin or 5 µg/ml erythromycin, when appropriate, as described previously (38).

*Escherichia coli* BL21 (λDE3)-Star cells (Invitrogen) were grown at 37 °C under standard conditions in either Luria-Bertani medium (LB; Thermo Fisher Scientific) or 2X YT medium (16 g/l tryptone, 10 g/l yeast extract, 5 g/l NaCl) supplemented with 100 µg/ml ampicillin, when appropriate.

#### 2.1.1 Construction of an HtrA–deficient mutant

An HtrA-deficient *T. forsythia* mutant was constructed using homologous recombination of a gene knockout cassette deleting WP_314949843.1 [Tanf_11420 (39)] as described previously (40). Positive clones were selected based on transferred erythromycin (Erm) resistance (Supplementary Figure S1). A detailed description of the construction of the knockout cassette is given in the Supplementary Material. In brief, ∼1 kbp up- and downstream homology regions were joined to the Erm-resistance gene by overlap-extension (OE) PCR and subsequently blunt-end cloned into the plasmid pJET1.2 (Thermo Fisher Scientific). The knock-out cassette was transformed into electrocompetent *T. forsythia* cells, which were regenerated overnight and plated on Erm-containing BHI selection plates. Single colonies were picked and used to inoculate BHI medium. Once bacterial growth was visible, genomic DNA was isolated (41) and the loss of the *htrA* gene was confirmed by PCR using Phusion High-Fidelity DNA polymerase (Thermo Fisher Scientific) for amplification.

#### 2.1.2 Back-complementation of the HrtA–deficient mutant

To confirm that the proteolytic activity observed on the tested substrates is solely attributable to HtrA, *T. forsythia* Δ*htrA* was complemented with the native gene, along with a Cat resistance gene (chloramphenicol acetyl transferase, *cat*; 650 bp) for selection (40). For the construction of *T. forsythia* Δ*htrA^+^*, the ∼1 kbp homologous upstream region plus the associated native *htrA* gene were joined to the *cat* gene using OE-PCR and subsequently blunt-end cloned into the plasmid pJET1.2. Using the artificially introduced restriction sites SphI and KpnI, the downstream homologous region was added, completing the reconstitution cassette (Supplementary Figure S1).

Oligonucleotides (Thermo Fisher Scientific) used for mutations at the *htrA* locus in *T. forsythia* are listed in Supplementary Table S1.

### 2.2 Bioinformatic prediction and classification of *T. forsythia* HtrA

Sequence alignment of *T. forsythia* WP_314949843.1 (Tanf_11420 (39) and orthologs from selected pathogens with experimentally proven HtrA activity was conducted. These pathogens included *P. gingivalis* (SJM20285.1), *Bacteroides fragilis* (OCR40173.1), *E. coli* K-12 substrain MC4100 (CDJ70742.1), *Shigella flexneri* Shi06HN006 (AIL38993.1), *Helicobacter pylori* (QFG75472.1), *Campylobacter jejuni* (WP_334204308.1), *Listeria monocytogenes* (WP_341777121.1), *B. anthracis* (GEU15401.1), *Borrelia burgdorferi* (WP_210376041.1), and *Legionella pneumophila* (GAN26037.1). Amino acid sequences were retrieved from NCBI (https://www.ncbi.nlm.nih.gov/) and aligned using clustalW (https://www.genome.jp/tools-bin/clustalw).

The protein family membership and domain assembly of HtrA from *T. forsythia* was analysed using InterPro (https://www.ebi.ac.uk/interpro/) and compared to that from *P. gingivalis* HtrA.

### 2.3 Production and purification of HtrA

#### 2.3.1 Expression of HtrA in *E. coli* BL21

The full-length HtrA protein, with a C-terminal His_10_-tag, was generated by PCR amplification of the *Tanf_11420* gene from genomic DNA of *T. forsythia* ATCC 43037. The PCR fragment was then cloned into pET16b *via* NcoI using primers 141fw (ATCACCCATGGGGGCAGTGACTTATAT GGTGAAGCACAATGCG) and 142rev (ATCACCCATGGTTAGTGATGATGATGATGATG TTCGGAGAGATTGATCGCGTAAAACTGTGTCC). Positive clones were confirmed by PCR screening, restriction digestion, and sequencing. The resulting plasmid, pET16b-*htrA*, was transformed into *E*. *coli* BL21 (λDE3)-Star cells (Invitrogen) and plated on LB agar plates containing 100 µg/ml of ampicillin. Single colonies were transferred to 5 ml of LB medium supplied with the antibiotic and grown overnight at 37 °C. This culture was then inoculated into five flasks containing 0.25 l of ampicillin-containing 2X YT medium and incubated at 37 °C with shaking at 200 rpm. Protein overexpression was induced at an OD_600_ ∼0.45 by adding isopropyl-β-d-thiogalactopyranoside (IPTG) to a final concentration of 0.6 mM. Cells were further incubated over night at 22 °C and 180 rpm, harvested by centrifugation at 5,000 rpm for 20 min at 4 °C, and the pellets were stored at −20 °C.

#### 2.3.2 Purification of rHtrA

Cell pellets were thawed on ice and resuspended in lysis buffer (50 mM sodium phosphate, pH 7.5, 300 mM NaCl, supplemented with 10 mM imidazole) containing 1 mg/ml lysozyme and DNAseI (Roche Applied Science), using 10 ml lysis buffer per gram of pellet (wet weight). The suspension was homogenized through ultrasonication with 6× 20 s pulses (Branson Sonifier 250; output 8, duty cycle 45%, 20 s breaks). After ultrasonication, the lysate was cleared by centrifugation and applied to a nickel NTA-affinity chromatography column (Qiagen) equilibrated with lysis buffer at a flow rate of 1.0 ml/min. The column was loaded with the supernatant fraction containing rHtrA-His_10_ and incubated for 20 min. Elution was performed in fractions of 1 ml using elution buffer (300 mM NaCl, 50 mM sodium phosphate) containing 50, 100, 150, 200, 250 mM imidazole, each step with 5 ml of elution buffer. Fractions from the different elution steps were pooled, and the presence of the desired protein was determined by 10% SDS-PAGE (42) upon Coomassie Brilliant Blue (CBB) staining for protein. Imidazole was removed with simultaneous buffer exchange to PBS (25 mM sodium phosphate buffer, pH 7.5) and the sample was concentrated to less than 1 ml using Amicon spin columns (MW cut-off 3,000 Da). The concentrated sample was further purified on a XK60/16 Superdex 200 size exclusion column (1.6 cm × 60 cm; GE-Healthcare) (SEC) run in 1× PBS at a flow rate of 0.25 ml/min (fraction size, 5 min); ∼15 mg of protein were loaded per run. Fractions containing HtrA-His_10_ were pooled based on to SDS-PAGE analysis and the protein concentration was determined by the Bradford assay (43).

#### 2.3.3 Endotoxin removal

For removal of endotoxin from the purified rHtrA-His_10_ sample intended for use in cell culture, Pierce High-Capacity Endotoxin Removal Spin Columns (Thermo Fisher Scientific) were employed according to the manufacturer's instructions. Briefly, prior to use, the spin column was washed with 0.2 M NaOH in 95% ethanol for 2 h, followed by 2 M NaCl and endotoxin-free water (Thermo Fisher Scientific). After equilibrating the resin with PBS, an aliquot of purified rHtrA-His_10_ in 1× PBS was added and incubated at room temperature for 1 h with gentle end-over-end mixing. Subsequently, the column was centrifuged at 5,000 rpm for 1 min to collect the sample. The endotoxin concentration in the sample was measured using the LAL Chromogenic Endotoxin Quantitation Kit (Thermo Fisher Scientific) according to the manufacturer's specifications. Endotoxin units (EU) were converted in mg/ml to calculate the effective concentration of endotoxin in the samples used for host cell stimulation (1 EU/ml is approximately 0.1–0.2 ng/ml).

### 2.4 *In vitro* cleavage assays of HtrA

#### 2.4.1 Casein zymogram

Bacterial extracts of *T. forsythia* ATCC 43037, the HtrA deficient mutant *T. forsythia* Δ*htrA*, the back-complemented mutant *T. forsythia* Δ*htrA*^+^, and rHtrA-His_10_ were analysed using a casein zymogram to assess proteolytic activity (30). For this purpose, a 10% non-reducing SDS gel containing 0.1% casein was prepared, and an equivalent of 1 ml of *T. forsythia* culture and 250 ng of rHtrA-His_10_ were loaded on the gel. After separation by SDS-PAGE, the gel was soaked in 2.5% Triton X-100 to remove SDS (for 30 min) and subsequently incubated in developing buffer (50 mM Tris/HCl, 100 mM NaCl, 5 mM CaCl_2_, pH 7,4), followed by overnight incubation at 37 °C to allow for casein cleavage. Subsequently, the gel was stained in 0.5% CBB in water for 2 h. Caseinolytic activity was visualized by cleared (bright) zones against blue background.

#### 2.4.2 E-cadherin cleavage

Recombinant E-cadherin (C-terminally His-tagged; rE-Cad; R&D Systems; dissolved in PBS at 100 μg/ml and stored at −70 °C) was incubated with rHtrA-His_10_ at a ratio of 1:1.5 (wt/wt), *i.e.*, 500 ng of rE-Cad and 750 ng of rHtrA-His_10_, over a duration of 16 h. Samples were collected at 0.5, 1, 2, 4, 8 and 16 h, and pure rE-Cad and rHtrA served as controls. The reaction mixtures were separated by 10% SDS-PAGE and transferred onto a nitrocellulose membrane (BioRad) using a Mini Trans-Blot Cell (Bio-Rad) for 2 h at 110 V and 350 mA. E-cadherin cleavage was detected using a rabbit α-E-cadherin antibody (Santa Cruz Biotechnology) diluted at 1:2500, which specifically recognizes the E-cadherin ectodomain. rHtrA-His10 and rE-Cad were detected using a mouse α-His antibody in combination with a donkey anti-mouse antibody labelled with IRDye680 (LI-COR Biosciences) and a rabbit α-E-Cad antibody in combination with a goat anti-rabbit antibody labelled with IRDye680 (LI-COR Biosciences), respectively. Protein bands were visualized at 800 nm using an Odyssey Infrared Imaging System (LI-COR Biosciences).

### 2.5 Infection of mammalian cell culture

#### 2.5.1 Cell lines

The U937 monocytic cell line (ATCC; referred to as U937 macrophages) was cultured in RPMI 1,640 medium supplemented with 10% (v/v) fetal bovine serum (FBS) and penicillin (100 U/ml)-streptomycin (100 µg/ml) (Pen-Strep) at 37 °C in a humidified atmosphere containing 5% CO_2_ (39). For differentiation into adherent macrophages, U937 macrophages were seeded at a density of 3 × 10^6^ cells/ml in a 6-well plate and treated with PMA (Sigma) at a concentration of 0.2 µg/ml for 72 h.

Primary human gingival fibroblasts (hGFBs) were isolated from the gingival tissue of periodontally and systemically healthy individuals. Gingival tissue was excised using a scalpel and placed into Dulbecco's Modified Eagle's Medium (DMEM; Invitrogen) supplemented with 10% FBS and Pen-Strep. The tissue was shredded into small pieces and incubated at 37 °C in a humidified atmosphere containing 5% CO_2_ to allow cell outgrowth (44–46). The protocol for tissue collection and cell isolation was approved by the Ethics Committee of the Medical University of Vienna (Protocol 1079/2019). For experiments involving hGFBs, cells were cultured up to a maximum of seven passages, and five donors of similar age were selected for the experiments. Cells were maintained in DMEM (Sigma-Aldrich) supplemented with 10% FBS and Pen-Strep at 37 °C in a humidified atmosphere containing 5% CO_2_.

#### 2.5.2 Set-up of bacterial stimuli

For infection studies, hGFBs and U937 macrophages were seeded into 24-well plates at a density of 5 × 10^4^ cells/well and 3 × 10^5^ cells/well, respectively, in 0.5 ml of the respective medium. Stimulation was performed at a multiplicity of infection (MOI) of 50 with viable *T. forsythia* wild-type, *T. forsythia* Δ*htrA*, and 10 ng/ml of rHtrA, respectively, diluted in the respective cell culture medium without FBS and antibiotics. Infections were terminated after 4 h and 24 h. Bacterial cell numbers were determined based on the correlation between OD_600_ values and colony forming units (CFU) per millilitre of culture using dilution plating and colony counting with three biological replicates and three technical replicates, each, as described previously (47). An OD_600_ of 1.0 corresponds to 3 × 10^8^ CFU of *T. forsythia* wild-type and *T. forsythia* Δ*htrA*.

#### 2.5.3 MTT cell viability assay

After 4 h and 24 h post-infection, 100 μl of MTT-reagent (5 mg/ml; Sigma-Aldrich) was added to each well. Following a 2 h incubation at 37 °C and 5% CO_2_, the medium was aspirated and 500 μl DMSO was added to each well to dissolve formazan crystals. Measurements were taken in quadruplicate. Specifically, 100 μl from each well were transferred into four different wells of a 96-well plate, and the absorbance was measured at 570 nm using the microplate reader Synergy HTX multiplate reader (BioTek).

#### 2.5.4 Gene expression of pro-inflammatory cytokines

Following infection, cell lysis, reverse transcription into cDNA, and qPCR were carried out using the TaqMan® Gene Expression Cells-to-CT™ kit (Ambion/Applied Biosystems) (47, 48). Reverse transcription was conducted using the Primus 96 advanced thermocycler (PEQLAB/VWR). Samples were incubated at 37 °C for one hour and subsequently at 95 °C for 5 min. qPCR was performed using StepOnePlus (Applied Biosystems), with a program consisting of initial denaturation at 95 °C for 10 min followed by 50 cycles at 95 °C for 15 s and annealing/extension at 60 °C for one minute. The target genes were amplified using the following TaqMan gene assays (all Applied Biosystems): IL-1β Hs01555410_m1, TNF-α, Hs99999043_m1; IL-6, Hs00985639_m1; IL-8, Hs00174103_m1; MCP-1, Hs00234140_m1; GAPDH, Hs99999905_m1. C_t_ values were determined for each gene and the expression of the target gene was calculated by the 2^−ΔΔCt^ method, where ΔΔC_t_ = (C_t_^target^−C_t_^GAPDH^) sample—(C_t_^target^−C_t_^GAPDH^) control. Cells that were not stimulated with bacteria served as controls.

#### 2.5.5 Quantification of secreted cytokines by ELISA

The concentration of the inflammatory mediators IL-1β, IL-6, IL-8, MCP-1, and TNF-α in conditioned media was determined using uncoated ELISA kits (Invitrogen) following the manufacturer's protocol. The sensitivity of the ELISA was 2 pg/ml for IL-1β, IL-6, and IL-8, 7 pg/ml for MCP-1, and 4 pg/ml for TNF-α.

The supernatant of hGFBs was measured undiluted, while the U937 supernatant was diluted 1:100 for 4 h of infection and 1:500 for 24 h of infection (48). Absorbance at 450 nm, with wavelength correction at 570 nm, was measured, and the concentrations were calculated against known cytokine standards using the Synergy HTX multiplate reader and Gen5 2.09 software (BioTek).

### 2.6 Statistical evaluation

The normal distribution of the data was assessed using the Kolmogorov-Smirnov test. When normal distribution was confirmed in all groups, differences between them were assessed using ANOVA for repeated measures and an LSD post-hoc test for pairwise comparison. If normal distribution was not found in at least one group, the Friedman test was employed, followed by the Wilcoxon signed-rank test for pairwise comparison. The level of cytokines in the conditioned media at 4 and 24 h post-stimulation was compared using the Wilcoxon signed-rank test. Data are presented as mean ± SEM, and differences were considered statistically significant at *p* < 0.05. All statistical analyses were conducted using IBM SPSS Statistics Version 24 (IBM Corporation).

## 3 Results

### 3.1 Identification and predicted domain assembly of *T. forsythia* HtrA

**Figure 1:**
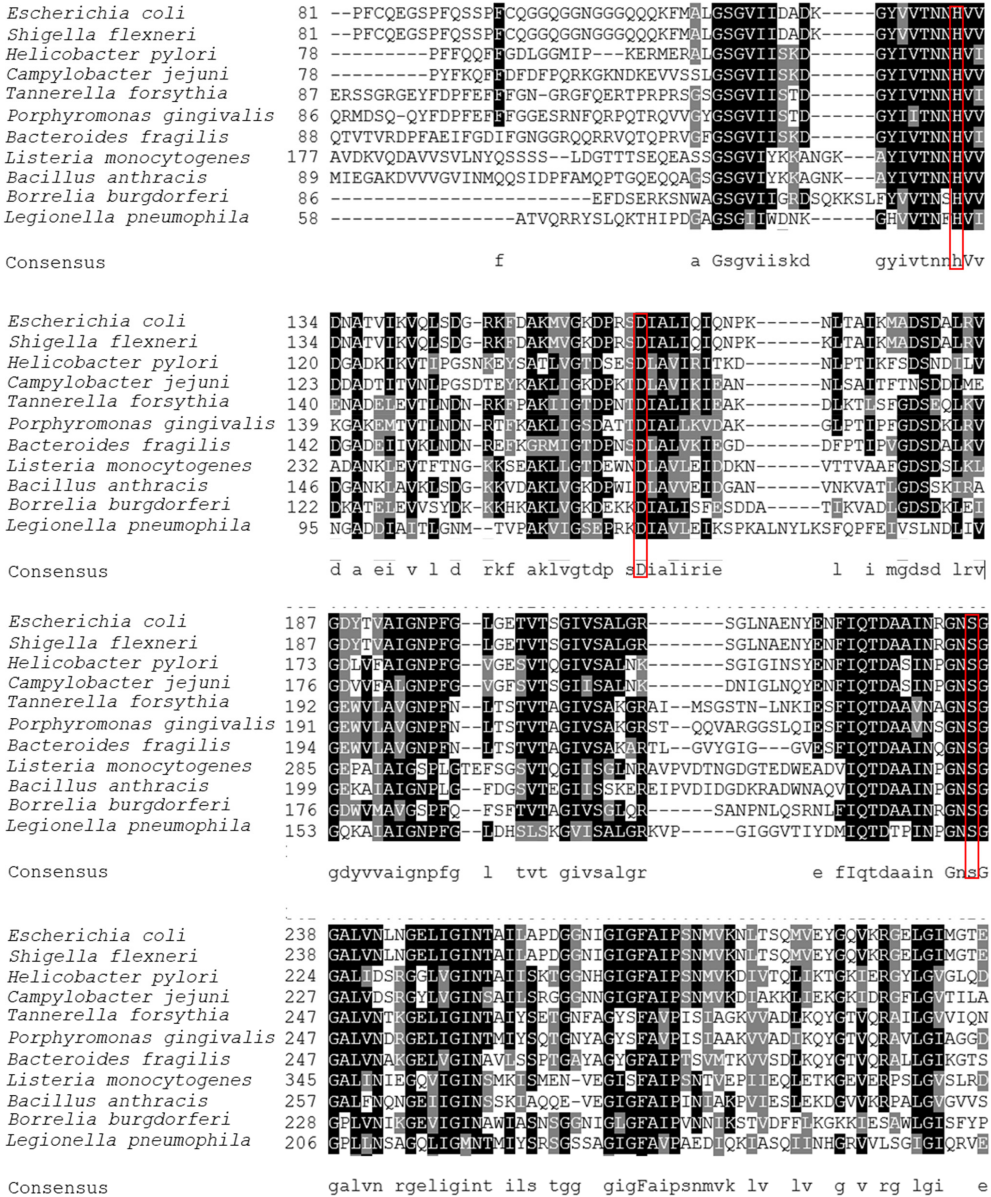
Multiple sequence alignment of HrtA protein orthologs from *Tannerella forsythia* (WP_314949843.1), *Porphyromonas gingivalis* (SJM20285.1), *Bacteroides fragilis* (OCR40173.1), *Escherichia coli* K-12 substrain MC4100 (CDJ70742.1), *Shigella flexneri* Shi06HN006 (AIL38993.1), *Helicobacter pylori* (QFG75472.1), *Campylobacter jejuni* (WP_334204308.1), *Listeria monocytogenes* (WP_341777121.1), *Bacillus anthracis* (GEU15401.1), *Borrelia burgdorferi* (WP_210376041.1), *Legionella pneumophila* (GAN26037.1). The multiple sequence alignment is displayed using the BoxShade program (https://junli.netlify.app/apps/boxshade/). Identical residues are shaded in black, similar residues are shaded in grey. Of note, the sequence alignment is shown only for the middle part of HtrA sequences, where the catalytic triad (His, Asp, Ser), boxed in red, is located (for *T. forsythia* HtrA, this corresponds to amino acids 87–307).

Tanf_11420 is annotated as 54.2-kDa HtrA protein (calculated molecular weight based on amino acid sequence), with high homology observed in its central protein region, comprising the Ser-His-Asp catalytic triad (corresponding to amino acids 87–307 in *T. forsythia* HtrA), to annotated HtrA protein family members from various pathogens (Figure 1).

InterPro analysis predicted a domain assembly for the *T. forsythia* 502-amino acid HtrA protein, consisting of a trypsin-like serine protease domain (spanning amino acid residues 66–290) followed by two PDZ domains-PDZ1 (279–397) and PDZ2 (406–485). The prediction for the N-terminus is inconclusive; a region spanning amino acids 7–29 is predicted to be embedded in the membrane, while amino acids 1–24 might also constitute a signal peptide. A comparison with the 498-amino acid long HtrA from *P. gingivalis* reveals a very similar result, predicting a trypsin-like serine protease domain (amino acids 63–290), PDZ1 (298–389) and PDZ2 (406–485), with the N-terminus potentially constituting either a signal peptide or a transmembrane domain.

### 3.2 Recombinant production and purification of *T. forsythia* HtrA

**Figure 2:**
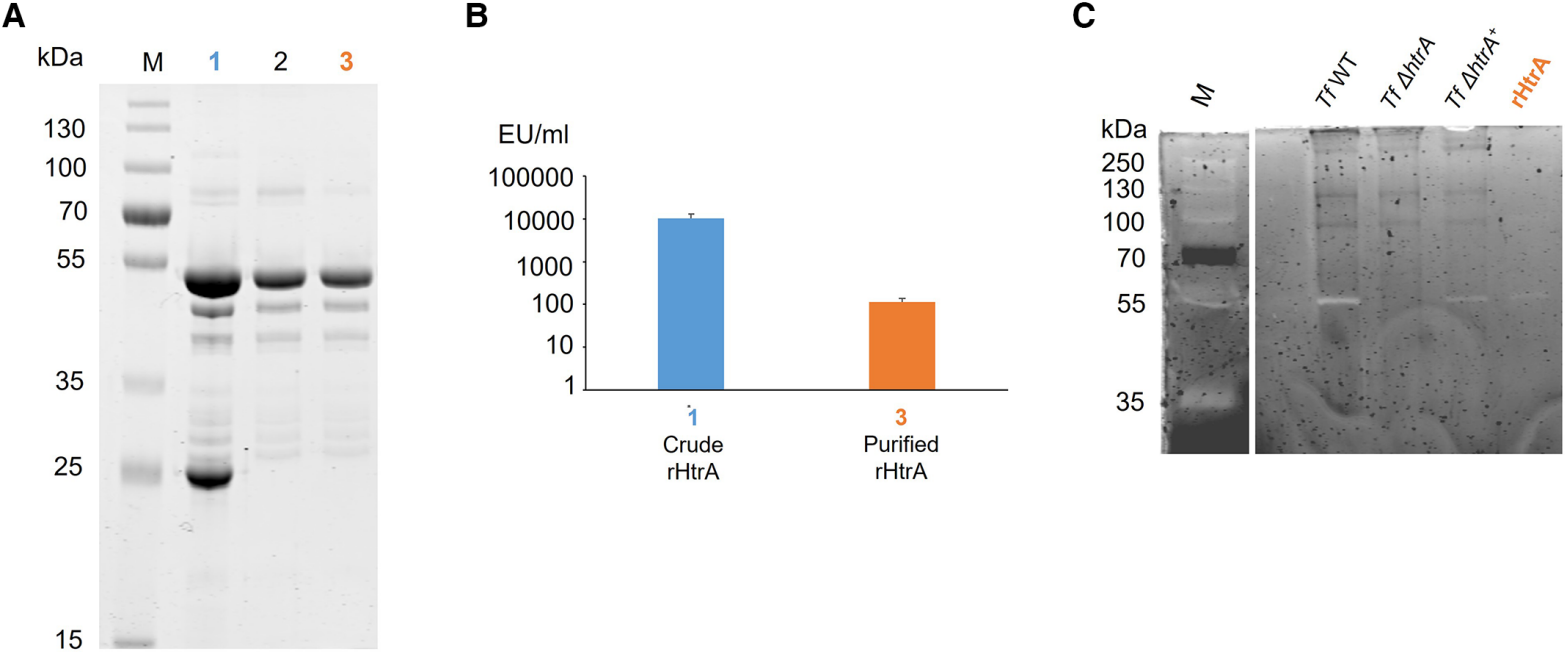
(**A**) SDS-PAGE analysis (10% gel) upon protein staining with CBB showing purification steps of rHtrA-His_10_, migrating around 54 kDa. (1) Crude extract from an *E. coli* overexpression culture, (2) rHtrA-His_10_ after purification *via* SEC, (3) rHtrA-His_10_ after endotoxin removal. M, Page Ruler Prestained Plus (Thermofisher). (**B**) Endotoxin quantification (EU/ml) in (1) a crude enzyme extract from the *E. coli* overexpression culture and (3) purified rHtrA after application on a polymyxin B column showing a 100-fold decreased endotoxin concentration. (**C**) Zymogram showing caseinolytic activity of *T. forsythia* wild-type in comparison to the *htrA* deletion mutant, the back-complemented mutant and rHtrA. A bright band around 55 kDa (*) corresponds to casein degradation by HtrA in all samples except for *T. forsythia* Δ*htrA*, confirming that the observed proteolytic effect is due solely to *T. forsythia* HtrA. M, Page Ruler Prestained Plus (Thermofisher).

Cloning of Tanf_11420 into pET16b followed by overexpression in *E. coli* BL21 cells and purification of rHtrA-His_10_ by nickel NTA affinity chromatography and SEC yielded in total 5 mg of rHtrA-His_10_ from 1 l of overexpression culture (Figure 2A), which was further utilized for analysis by casein zymography and an *in vitro* E-cadherin cleavage assay.

Before infecting hGFBs and U937 macrophages, endotoxin was removed from the HtrA-His_10_ preparation using a polymyxin B column. The subsequent LAL test revealed a 100-fold reduction in endotoxin level in the final purified protein solution (2 mg in total) compared to the sample after SEC (Figure 2B). Consequently, the endotoxin concentration during the stimulation of host cells did not exceed 1.6 pg/ml. Notably, our previous stimulation studies of U937 macrophages (49) and gingival fibroblasts (50) with LPS from *T. forsythia* and *P. gingivalis*, respectively, at a concentration of 10 ng/ml did not induce the production of inflammatory mediators. Thus, for the present study, the LPS concentration in the samples is negligible.

### 3.3 Enzymatic activity of HtrA

#### 3.3.1 Caseinolytic activity

The proteolytic activity of native HtrA (produced by *T. forsythia* cells) and recombinant HtrA (rHtrA-His_10_) was analysed alongside the HtrA-deficient mutant, using a casein zymogram (Figure 2C). The presence of caseinolytic activity was indicated by a bright band migrating at ∼55 kDa in crude cell extracts of *T. forsythia* wild-type, the reconstituted mutant *T. forsythia* Δ*htrA*^+^, and the recombinant enzyme. As expected, no activity was observed in *T. forsythia* Δ*htrA*. Remarkably, proteolytic activity could be fully restored in the mutant complemented with the native *htrA* gene (*T. forsythia* Δ*htrA*^+^), indicating that Tanf_11420 is indeed the only source of caseinolytic activity in *T. forsythia*.

#### 3.3.2 E-cadherin cleavage

**Figure 3:**
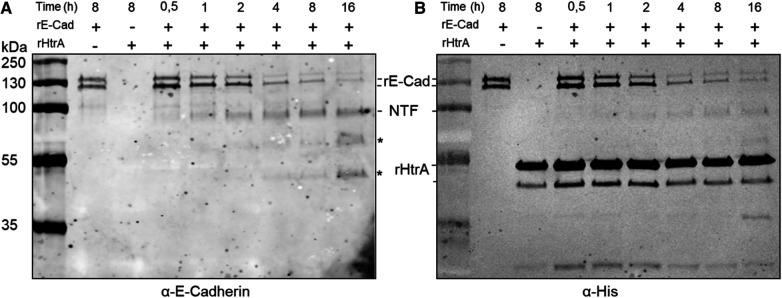
Time course (0.5 h–16 h) of recombinant, E-cadherin (rE-Cad, C-terminally His-tagged) cleavage by recombinant *T. forsythia* HtrA (rHtrA-His_10_) as determined by Western-blot analysis using antibodies (α) directed against rE-Cad and His-tag. (**A**) Cleavage of E-cadherin (125 kDa; rE-Cad) by rHtrA-His_10_ yields an 85 kDa ectodomain cleavage product (NTF, N-terminal fragment) and a 40 kDa C-terminal fragment (CTF), with increased rE-Cad degradation visible upon prolonged incubation visualized by decreasing intensity of the rE-Cad bands and increasing intensity of the NTF-band. Of note, commercial rE-Cad has a bi-banded appearance on the gel. * indicates potential N-terminal cleavage products. (**B**) Detection of rHtrA and rE-Cad *via* the His-tag, indicating stability of rHrtA-His_10_ over the time course (recombinant *T. forsythia* HtrA has a bi-banded appearance on the gel) and confirming rE-Cad degradation over time. rHtrA-His_10_, recombinant, C-terminally His_10_-tagged *T. forsythia* enzyme; rE-Cad, commercial, recombinant, C-terminally His-tagged E-cadherin; NTF, N-terminal rE-Cad ectodomain.

To analyse whether *T. forsythia* HtrA acts as an E-cadherin protease, rHtrA-His_10_ was incubated with rE-Cad for a duration of 16 h. Cleavage of the 125 kDa, C-terminally His-tagged rE-Cad by rHtrA resulted in an 85 kDa, N-terminal ectodomain cleavage product (detectable by both anti-His and anti-E-cadherin ectodomain antibodies) and a 40 kDa N-terminal, transmembrane/intracellular cleavage product (not detectable by the rE-Cad antibody); degradation was visible already after 0.5 h of incubation (Figure 3A). An anti-His Western-blot confirmed that rE-Cad degradation by rHtrA-His_10_ increased over time, with the most degradation visible after 16 h of incubation; simultaneously stability of the enzyme over the time course was demonstrated (Figure 3B).

### 3.4 Influence of *T. forsythia* wild-type, HtrA-deficient mutant and rHtrA on the viability of hGFBs and U937 macrophages

**Figure 4:**
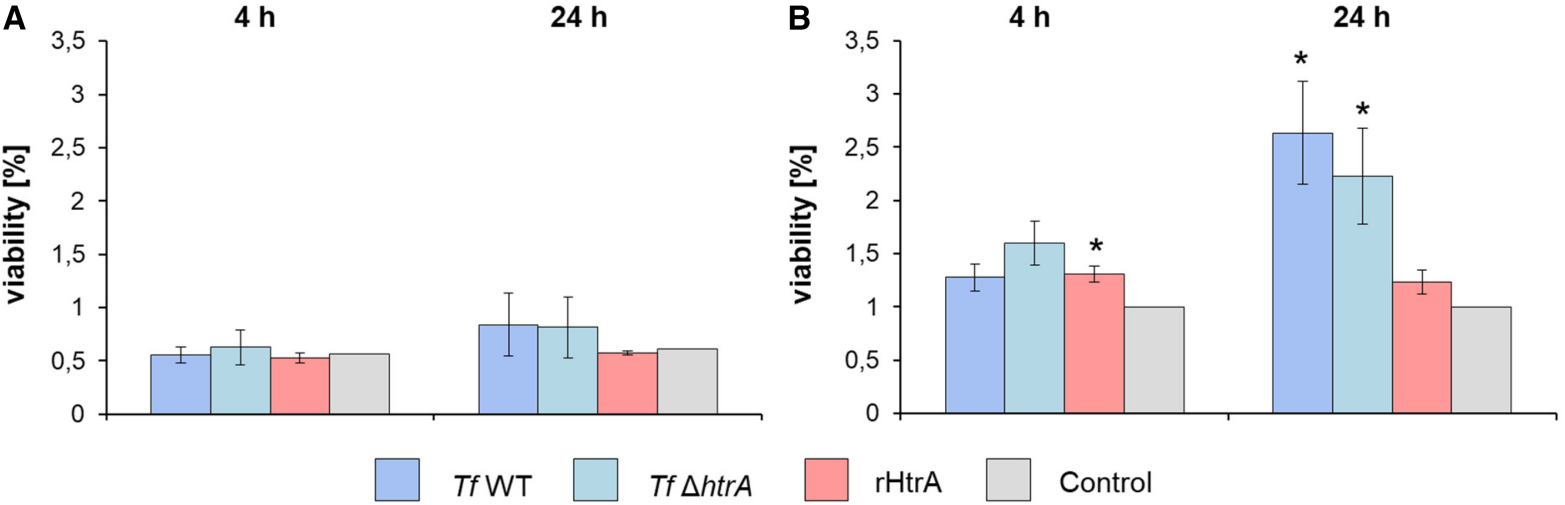
Viability determined by an MTT assay of (**A**) hGFBs and (**B**) U937 macrophages after infection with *T. forsythia* ATCC 43037 (*Tf* WT) and *T. forsythia* ATCC 43037 Δ*htrA* (*Tf* Δ*htrA*) at an MOI of 50 and with 10 ng/ml rHtrA, respectively. Each bar represents mean viability ± SEM; *indicates significant differences to the untreated control. Significance was tested through ANOVA (*P* ≤ 0.05).

The impact of infecting hGFBs and U937 macrophages with *T. forsythia* cells (*T. forsythia* wild-type and *T. forsythia* Δ*htrA*) and rHtrA, respectively, on the viability of the two cell types was assessed using an MTT assay after both 4 h and 24 h of exposure, as depicted in Figure 4. In hGFBs, no significant effect on viability was observed for any stimulus at either time point (Figure 4A). The viability of U937 macrophages was enhanced by all stimuli after both 4 and 24 h of infection. A statistically significant increase in U937 viability was noted only for treatment with rHtrA after 4 h and infection with both *T. forsythia* species after 24 h (Figure 4B).

### 3.5 Response of hGFBs to infection with *T. forsythia* wild-type, HtrA-deficient mutant and rHtrA

**Figure 5:**
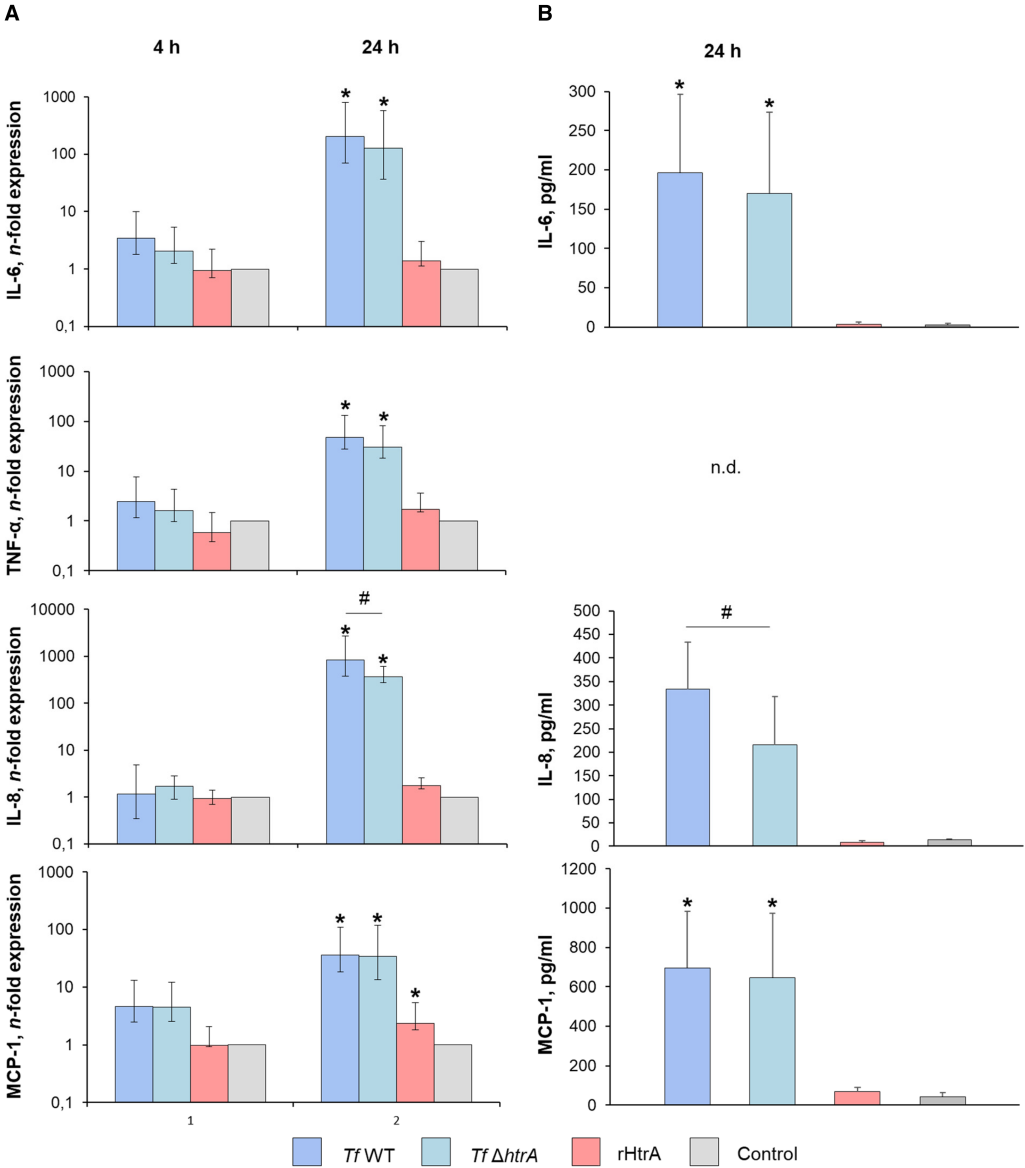
(**A**) Gene expression and (**B**) secretion of pro-inflammatory cytokines by hGFBs after infection with *T. forsythia* ATCC 43037 (*Tf* WT) and *T. forsythia* Δ*htrA* (*Tf* Δ*htrA*) at MOI of 50 or with 10 ng/ml rHtrA. Gene expression of IL-6, TNF-α, IL-8 and MCP-1 is shown as n-fold expression (log scale) compared to the untreated control. Concentrations of the corresponding proteins are plotted as pg/ml. For TNF-α, no cytokine secretion was detectable at both time points. Data are shown as mean ± SEM; * indicates statistically a significant increase compared to the control, # indicates a significant difference between groups (*P* ≤ 0.05) as determined by the Friedman test followed by the *post hoc* Wilcoxon test for pairwise comparison. n.d., not detectable.

Figure 5 illustrates the impact of infecting hGFBs with *T. forsythia* wild-type and *T. forsythia* Δ*htrA* at an MOI of 50, was well as with 10 ng/ml rHtrA, on the production of pro-inflammatory cytokines IL-6, TNF-α, IL-8 and MCP-1, as measured by qPCR and ELISA. After 4 h, no effect of any stimulus on the gene expression of pro-inflammatory mediators was observed (Figure 5A). The protein levels of most cytokines after 4 h of stimulation was below the detection limit of the ELISA (data not shown).

After 24 h of infection, both *T. forsythia* stimuli significantly upregulated gene expression of IL-6, TNF-α, IL-8, and MCP-1. Notably, the *T. forsythia* Δ*htrA* mutant exhibited a significantly lesser effect on gene expression of IL-8 compared to the wild-type. Stimulation with rHtrA resulted in a significant enhancement of MCP-1 expression only; otherwise, the gene expression levels following treatment with the recombinant enzyme did not differ from those of the untreated control (Figure 5B). Correspondingly, IL-6, IL-8 and MCP-1 levels in the cell culture supernatant were markedly elevated after bacterial infection, although statistically significant differences were observed only for IL-6 and MCP-1 (Figure 5B). Additionally, there was a significantly lower IL-8 level upon infection with the Δ*htrA* deletion mutant compared to the parent wild-type strain. No significant effect of rHtrA on the production of IL-6, IL-8 or MCP-1 by hGFBs was observed. TNF-α levels were below the detection levels after 24 h of stimulation (Figure 5B).

### 3.6 Response of U937 macrophages to infection with *T. forsythia* wild-type, HtrA-deficient mutant and rHtrA

**Figure 6:**
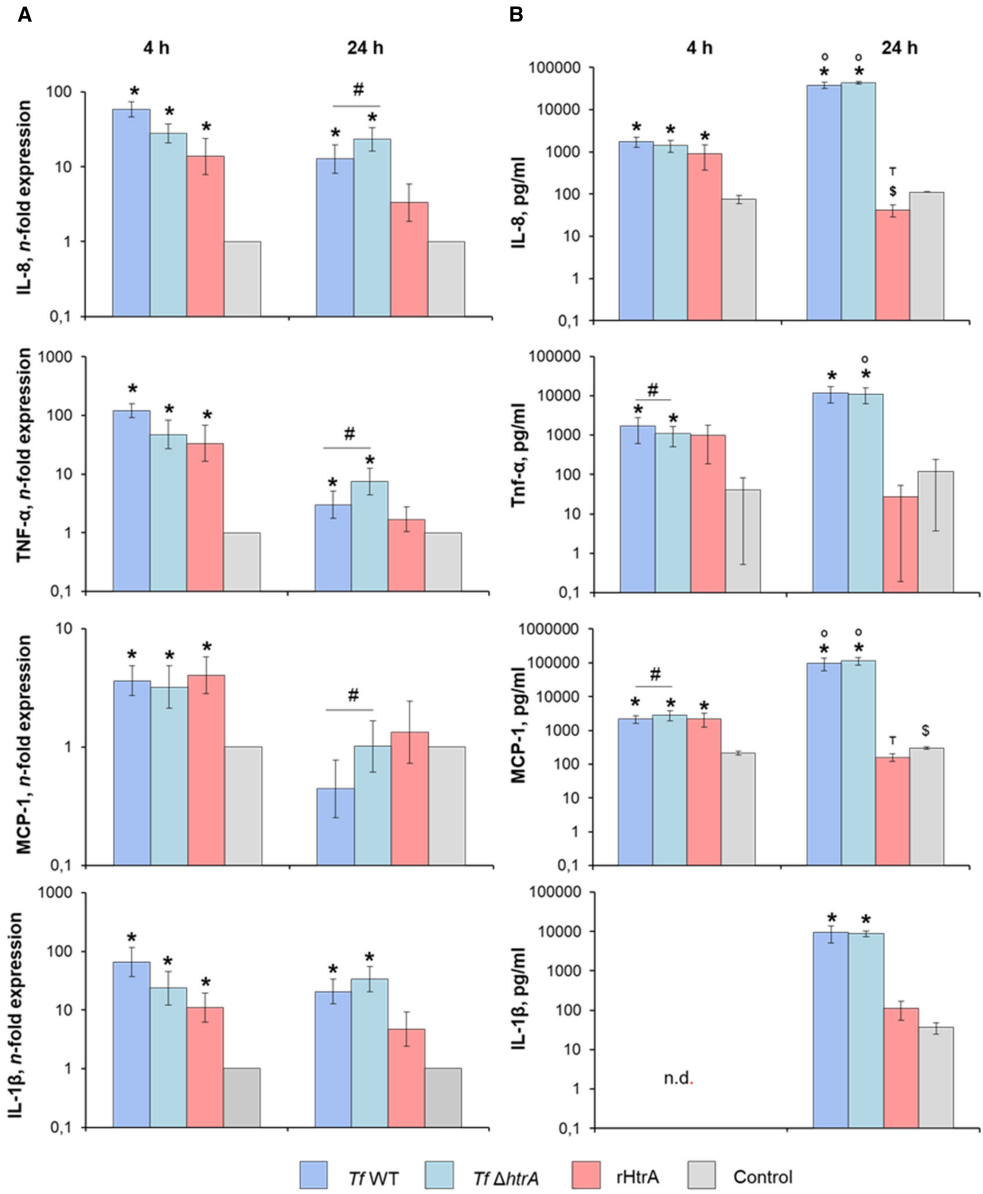
(**A**) Gene expression and (**B**) secretion of pro-inflammatory cytokines by U937 macrophages after infection with *T. forsythia* ATCC 43037 (*Tf* WT) and *T. forsythia* ATCC 43037 Δ*htrA* (*Tf* Δ*htrA*) at an MOI of 50 or with 10 ng/ml rHtrA. Gene expression of IL-6, TNF-α, IL-8 and MCP-1 is shown as n-fold expression (log scale) compared to the untreated control. Concentrations of the corresponding proteins are plotted as pg/ml. Data are shown as mean ± SEM; * indicates a statistically significant increase compared to the control, $ indicates a statistically significant decrease compared to the control, # indicates a significant difference between group, ° indicates significantly higher cytokine levels at 24 h post-infection, т indicates significantly lower cytokines levels at 24 h post-infection when compared to the earlier timepoint (*P* ≤ 0.05). Statistically significant differences were as determined by the Friedman test followed by the *post hoc* Wilcoxon test for pairwise comparison. n.d., not detectable.

Gene expression and protein production of various pro-inflammatory mediators in U937 macrophages stimulated with *T. forsythia* wild-type and *T. forsythia* Δ*htrA* at an MOI of 50, as well as with 10 ng/ml of rHtrA, are presented in Figure 6. At 4 h post-infection, all stimuli induced significantly higher gene expression levels of IL-8, TNF-α, MCP-1, and IL-1β (Figure 6A). The production of IL-8, TNF-α, and MCP-1 protein was significantly increased by all stimuli except for TNF-α upon rHtrA treatment (Figure 6B). Although both *T. forsythia* stimuli induced the production of pro-inflammatory mediators of a similar magnitude, *T. forsythia* Δ*htrA* infection resulted in significantly higher levels of TNF-α and significantly lower levels of MCP-1 compared to infection with the *T. forsythia* wild-type.

After 24 h of stimulation, both *T. forsythia* stimuli induced a significant increase in the gene expression of IL-8, TNF-α, and IL-1β, whereas that of MCP-1 was unaffected by the bacteria (Figure 6A). Furthermore, the gene expression levels of IL-8, TNF-α, and MCP-1 upon infection with *T. forsythia* Δ*htrA* were significantly higher compared to infection with *T. forsythia* wild-type. No significant effect of rHtrA on the gene expression of any pro-inflammatory mediator was observed. On the protein level, significantly higher production of all pro-inflammatory mediators at 24 h post-infection with both *T. forsythia* wild-type and Δ*htrA* was observed, with no difference between wild-type and the mutant (Figure 6B). The protein levels of TNF-α, MCP-1 and IL-1β upon the treatment with rHtrA for 24 h equalled those of the uninfected controls, whereas the level of IL-8 was significantly decreased by rHtrA infection compared to the control. Moreover, the levels of IL-8 and MCP-1 in the conditioned media of U937 macrophages treated with rHtrA for 24 h were significantly lower than those of U937 macrophages treated with rHtrA for 4 h.

## 4 Discussion

HtrA serine proteases are widely distributed among prokaryotic and eukaryotic species. Through their participation in protein quality control and cellular stress responses, they are linked to several clinical illnesses, including bacterial infections, cancer, and neurodegenerative diseases (20). There is increasing evidence that for bacterial pathogens, HtrAs are vital for establishing infections and survival under stress conditions, such as functioning as chaperones and signalling to the host immune system (19). Despite low overall sequence identity among HtrA family members, they have a conserved catalytic triad and can be relatively identified using bioinformatic tools. This study identified and characterized a predicted HtrA protease from the oral pathogen *T. forsythia* ATCC 43037 [WP_314949843; Tanf_11420 (39)] (Figure 1) and aimed to assess its potential contribution to bacterial virulence in a combined enzymatic and cell infection approach.

As a member of the red complex, *T. forsythia* is intimately linked to the establishment of periodontal infections (8, 9), with the full repertoire of its virulence factors still unknown. In order to infect host tissue, pathogenic bacteria must overcome the epithelial barrier by destroying cell adhesion molecules like E-cadherin (51). Ectodomain cleavage from E-cadherin occurs frequently and is an important step in the pathogenesis of inflammatory responses or neoplastic transformation (52). It is known that HtrA plays a role in this process due to its proteolytic activity, and it is conceivable to assume that this is of relevance for the establishment of periodontal infections. We produced *T. forsythia* HtrA recombinantly in *E. coli* cells (Figure 2A) and tested its proteolytic activity *in vitro* on an artificial (casein) and extracellular (E-cadherin) substrate, in comparison to the *T. forsythia* wild-type bacterium and an HtrA-deficient mutant created within the frame of this study (*T. forsythia* Δ*htrA*). Shedding of E-cadherin was proven for the DegP and DegQ homologs of several Gram-negative pathogens like *H. pylori*, enteropathogenic *E. coli* (EPEC), *Yersinia enterocolitica* and *Proteus mirabilis* (53).

The only HtrA protease from a periodontal pathogen investigated so far is that from *P. gingivalis*, for which involvement in oxidative and temperature stress responses has been reported, such as by stabilizing gingipains under these conditions (36), as well as in cell invasion (37). In this study, we demonstrated caseinolytic activity of *T. forsythia* wild-type, complemented *T. forsythia* Δ*htrA^+^* mutant as well as the recombinant enzyme (rHtrA-His_10_), with *T. forsythia* Δ*htrA* serving as a negative control (Figure 2C). Furthermore, degradation of commercial, recombinant E-cadherin (rE-Cad) by the recombinant enzyme was unambiguously proven in a time-course experiment and Western-blot analysis, showing an increase of the characteristic 85 kDa ectodomain fragment resulting from cleavage of the 125 kDa full-size rE-Cad over time (Figure 4). This corresponds to studies in *H. pylori*, demonstrating that HtrA cleaves off the ectodomain of E-cadherin (54). Notably, an HtrA ortholog was also found in another inhabitant of the oral cavity, the Gram-positive *Streptococcus mutans*, where enzyme expression was linked to the maturation of extracellular/surface associated proteins and biofilm formation (55).

To analyse the effect of *T. forsythia* HtrA on mammalian cells, we have chosen hGFBs, which are matrix-producing cells of the connective tissue ensuring the structural integrity of the gingiva (56), and macrophages, specialized immune cells capable of recognizing and processing antigens, including proteins (57). Importantly, the viability of hGFBs and U937 macrophages was maintained upon simulation with *T. forsythia* wild-type and Δ*htrA* mutant as well as endotoxin-free rHtrA (Figure 2B), based on an MTT assay (Figure 4), widely recognized as a measure of proliferating, viable cells (58). In hGFBs, no significant effect of either bacteria or rHtrA on viability was observed (Figure 4A), whereas the viability of U937 macrophages was increased by rHtrA after 4 h and by *T. forsythia* wild-type after 24 h post-infection. The ability of bacteria to enhance the viability of macrophages but not hGFBs was already observed in our previous study (47). While there was no significant difference between wild-type and HtrA-deficient *T. forsythia* regarding host cell viability, rHtrA significantly enhanced the viability of macrophages at the early phase of infection (4 h). This might indicate that HtrA *per se* can potentially signal to host cells (Figure 4B). Further studies are required to verify this hypothesis.

Regarding the potential contribution of HtrA to *T. forsythia’s* ability to activate host cells, two interesting findings emerged. Firstly, rHtrA induced an inflammatory response only in U937 macrophages, not in hGFBs. Secondly, the production of pro-inflammatory mediators by rHtrA-treated U937 macrophages was markedly higher after 4 h of infection than after 24 h. Moreover, after 24 h, the level of IL-8 in the supernatant of rHtrA-treated U937 macrophages was significantly lower than that in untreated cells. The differences between hGFBs and macrophages in their response to rHtrA could be explained by the different nature of these cells. It is plausible to assume that U937 macrophages recognize and process rHtrA as an exogenous antigen (57), resulting in their transient activation by this protein. Gingival fibroblasts are connective tissue cells of mesenchymal origin, involved in soft-tissue turnover and homeostasis maintenance (56, 59). They express different types of pattern recognition receptors and can be activated by different bacterial structures, like lipopolysaccharide (LPS) (60), but have no known specific receptor for rHtrA. The decreased content of pro-inflammatory mediators after 24 vs. 4 h could be explained if we assume that rHtrA possesses proteolytic activity also towards these mediators. We have observed that rHtrA cleaves casein and E-cadherin (Figures 2C, 3), but if it also applies to pro-inflammatory mediators, remains to be investigated.

To further elucidate the role of HtrA in the virulence of *T. forsythia*, we investigated how deletion of the *htrA* gene influences the response of hGFBs and U937-macrophages to this bacterium. However, our data regarding this question were inconclusive; in some cases, *T. forsythia* Δ*htrA* induced higher and, in some cases, lower responses than the wild-type species. Nevertheless, the magnitude of host cells response to both wild-type and HtrA-deficient *T. forsythia* was about the same, leading to the conclusion that HtrA does not play an essential role in the host response to *T. forsythia*. At first glance, this contradicts the observed ability of rHtrA to induce a transient activation in macrophages. However, it should be considered that *T. forsythia* expresses several factors capable of activating an inflammatory response in host cells, such as LPS (49) and OMVs (39), and their contribution might be more essential than that of HtrA. Furthermore, the cellular location of HtrA in *T. forsythia* still needs to be investigated. According to the current state of knowledge the enzyme is cargo of *T. forsythia’s* OMVs (39) in which it might be shuttled to its destination(s). This previous finding supports the presence of an N-terminal transmembrane domain in *T. forsythia* HtrA, predicted by InterPro with a higher score than a signal peptide, as revealed in this study, since anchoring of HtrA to the bacterial cell surface is regarded as an essential step in OMV transport and host invasion (22).

A limitation of this study lies in its *in-vitro* nature. We utilized only a single type of host cells to examine the host response and infected it with a single bacterial species or protein. In future, studies, the impact of HtrA should be further investigated in epithelial cells, which serve a barrier function and hinder bacterial invasion into gingival tissue (61).

In conclusion, *T. forsythia* HtrA does not significantly contribute to the pro-inflammatory immune response of hGFBs and U937 macrophages towards the bacterium. However, since the protease can cleave E-cadherin, it likely plays a more prominent role in bacterial cell invasion. The deletion mutant *T. forsythia* Δ*htrA* exhibited reduced viability and was more challenging to cultivate compared to its parental strain, suggesting a vital function in responding to environmental stresses and thereby in the persistence of the bacterium in its ecological niche. Considering that HtrA is a prominent cargo of *T. forsythia’s* OMVs (39) it is conceivable that the protein still contributes to *Tannerella’s* virulence potential, necessitating further studies to address these points.

## Data Availability

The raw data supporting the conclusions article will be made available by the authors, without undue reservation.
