# Supplementary material for: Investigating the role of a Tannerella forsythia HtrA protease in host protein degradation and inflammatory response
Source: Front Oral Health. 2024 Jul 5;5:1425937. doi: 10.3389/froh.2024.1425937 (PMC11257890; doi:10.3389/froh.2024.1425937)
Supplement: Supplementary file 1 [file Datasheet1.pdf]

*Supplementary Material*

# Investigating the role of a *Tannerella forsythia* HtrA protease in host protein degradation and inflammatory response

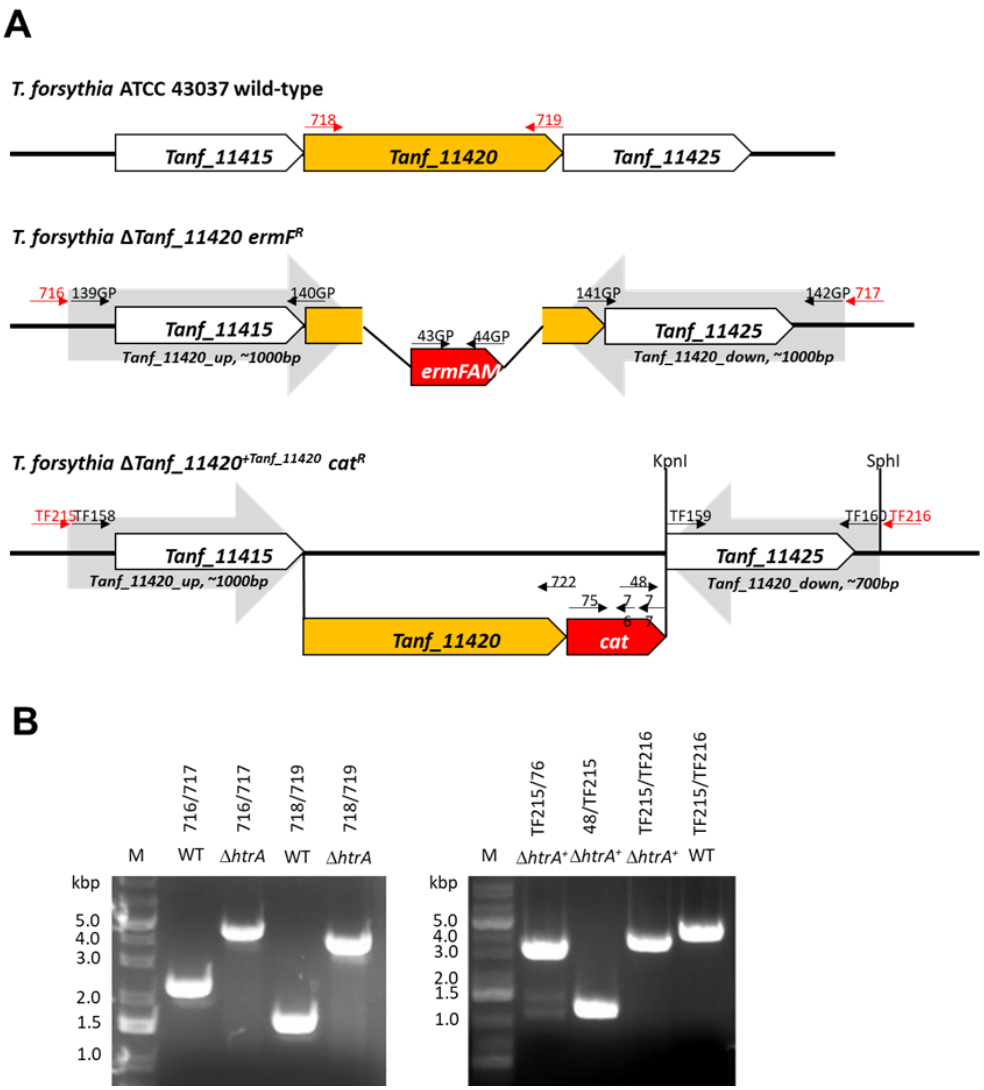


**Supplementary FIGURE S1 Strategy for the generation of a *T. forsythia* ATCC 43037 mutant at the *Tanf_11420* locus and confirmation by PCR. (A)** The genomic organization of the *Tanf_11420* locus is shown for the parent strain *T. forsythia* ATCC 43037 (wild-type), the Δ*Tanf_11420* mutant and the reconstituted mutant Δ*Tanf_11420*$^{+}$. Black coloured arrows represent primers used for PCR amplification of genes and homologous regions, red coloured primers represent those used to screen for correct integration of the knock-out and reconstitution cassettes; restriction sites used for cloning are indicated (not drawn to scale). **(B)** Agarose gel electrophoresis (left) confirmed the *T. forsythia* ATCC 43037 wild-type and the deletion of *Tanf_11420* with simultaneous integration of the *ermF* cassette using the upstream primers 718/719 (2198-bp product) and downstream primers 716/717 (4386-bp product) with genomic DNA of *T. forsythia* ATCC 43037 wild-type and the Δ*Tanf_011420* mutant, respectively, serving as a template. Agarose gel electrophoresis (right) confirmed the reconstitution of the deleted *Tanf_11420* gene using upstream primers 48/215 (1174 bp) on genomic DNA form Δ*Tanf_11420*$^{+}$ in comparison to the Δ*Tanf_11420* mutant, yielding a 2875-bp fragment using the primer pair 215/76. The result was verified with primers 215/216, yielding a 3370-bp product for *T. forsythia* ATCC 43037 wild-type and a 4021-bp product for Δ*Tanf_11420*$^{+}$. O´Gene Ruler 1 kb Plus DNA Ladder (Thermo Fisher Scientific) was used as a gene ladder.

**Supplementary Table S1**: Oligonucleotide primers for the construction of a *T. forsythia* Δ*htrA* deletion mutant and its back-complementation (*T. forsythia* $\Delta htrA^{+}$). Restriction sites are underscored. Lowercase letters indicate artificially introduced bases for restriction endonuclease digestion.

| **Primer** | **Sequence (5'-3')** |
|---|---|
| Construction of Δ*htrA* mutant | |
| 716 | ATCATGCGTTCCGCTCACTG |
| 717 | TCGGGTAGGCTAAGTCCTTG |
| 718 | ATGAAAACAATGTGGAAAAAAGG |
| 719 | TTATTCGGAGAGATTGATC |
| 139GP | ATCACGGATCCTCGCAGTGACTTATATGGTGAAGCACAATGCG |
| 140GP | ATCACCTCGAGTTATTCGGAGAGATTGATCGCGTAAAACTG |
| 141GP | ATCACCCATGGGGCAGTGACTTATATGGTGAAGCACAATGCG |
| 142GP | ATCACCCATGGTTAGTGATGATGATGATGATGTTCGGAGA<br>GATTGATCGCGTAAAACTGTGTCC |
| 43GP | CCCGGGGGAGGTACCCCCGATAGCTTC |
| 44GP | CCCGGGGGCTAGAGGATCCCCGAAGC |
| Reconstitution of Δ*htrA* mutant | |
| 48 | GTCAGATAGGCCTAATGACTGGC |
| 75 | ATGAACTTTAATAAAATTGATTTAGACAATTGG |
| 76 | TTATAAAAGCCAGTCATTAGGCCTATCTGAC |
| 77 | aatcaGCATGCGGTACCTTATAAAAGCCAGTCATTAGGCCTATCTGAC |
| 722 | CCAATTGTCTAAATCAATTTTATTAAAGTTCATTTATTCGGAGAGATTGATCG |
| TF158 | TTGATAAACAGCGAAGATGCGACAGC |
| TF159 | aatcaGGTACCACTCATCGGTCGGTGAGATTGTTATAG |
| TF160 | aatcaGCATGCCCGGAAATGCACTGCACGTC |
| TF215 | GATACGATGGCTAATGTGAACGG |
| TF216 | GGGTGACAATGCTCTATCTGACG |
